# CLC-Pred Synergy: Web Application for Predicting Pairwise Drug Combinations with Synergistic Activity Against NCI60 Cancer Cell Lines

**DOI:** 10.3390/ijms27125208

**Published:** 2026-06-09

**Authors:** Vladislav S. Sukhachev, Sergey M. Ivanov, Anastasia V. Rudik, Arseny R. Dublin, Dmitry A. Filimonov, Alexey A. Lagunin, Vladimir V. Poroikov

**Affiliations:** 1 Department of Bioinformatics, Institute of Biomedical Chemistry, Moscow 119121, Russia; withstanding@yandex.ru (V.S.S.); rudik_anastassia@mail.ru (A.V.R.); dmitry.filimonov@ibmc.msk.ru (D.A.F.); alexey.lagunin@ibmc.msk.ru (A.A.L.); vladimir.poroikov@ibmc.msk.ru (V.V.P.); 2 Department of Bioinformatics, Pirogov Russian National Research Medical University, Moscow 117513, Russia; dublin.arseny@yandex.ru

**Keywords:** anti-cancer, drug combination, synergy, NCI-ALMANAC, cell lines, structure-activity relationships, web application

## Abstract

Pharmacological intervention is central to cancer therapy, but overcoming drug resistance is limited by severe adverse effects. Rational drug combinations can improve efficacy and reduce toxicity, yet identifying effective pairs is challenging due to the vast combinatorial space. Structure-activity relationship (SAR) modeling offers an in silico approach to predict drug synergy. To create SAR models, training sets were built from the NCI-ALMANAC database, containing cytotoxicity profiles of ~5000 drug pairs per NCI-60 cell line. SAR models were developed using the PASS DDI (Prediction of Activity Spectra for Substances with the Drug-Drug Interaction) platform, which encodes chemical structures into PoSMNA (Pairs of Substances Multilevel Neighborhoods of Atoms) descriptors and applies a Bayesian-like algorithm to uncover structure–activity patterns. Model performance was assessed using leave-one-out compounds out cross-validation (LOO CO CV), with predictive accuracy quantified by the area under the ROC curve (AUC). A total of 104 SAR models demonstrated robust predictive performance, with AUC values exceeding 0.7 (mean AUC = 0.75), enabling the prediction of synergistic effects for compound combinations across 45 cancer cell lines. These models have been implemented in the CLC-Pred Synergy web application, providing a practical tool for in silico screening of anticancer drug combinations.

## 1. Introduction

Cancer is a malignant disease that causes millions of deaths each year and poses a significant obstacle to increasing life expectancy in the 21st century [1]. Chemotherapy is the conventional treatment to eliminate cancer cells or inhibit their proliferation. The challenge of effective treatment is overcoming cancer drug resistance and reducing the risk of serious adverse effects.

Synergistic drug combinations are one of the approaches to achieve these clinical benefits. First, combination therapy has been shown to have the potential to overcome resistance and enhance the response to existing drugs by addressing the issue of tumor heterogeneity and avoiding the selective pressure of a single drug. Second, the dose of each drug is smaller than what is typically used in monotherapy, which is why drug-induced adverse effects can be minimized [2]. Third, despite the lower dosage of each drug, the synergistic nature of their interaction could potentially provide a resulting therapeutic outcome greater than the expected additive effect [3]. Taking into account the above-mentioned factors, the discovery of effective combinations is needed.

The main principle underlying identification of effective combinations is related to the comparison of the experimentally measured effect of drug combination, e.g., growth fraction or percent of inhibition for the cell line, with expected effect of non-interaction (the null hypothesis) [4]. The value of the expected effect (y_e_) is calculated using data from dose-response curves for individual compounds. Four approaches are commonly used for this purpose: the Highest Single Agent model (HSA), the Bliss independence model (Bliss), the Loewe additivity model (Loewe), and the Zero Interaction Potency (ZIP) [4].

The expected effect in HSA model is calculated as the effect of the highest monotherapy effect: y_e_ = max(y_1_, y_2_), where y_1_ and y_2_ are effects of individual compounds.

The expected effect in Bliss model is calculated as the effect that would be achieved if the two drugs are acting independently of the phenotype: y_e_ = y_1_ + y_2_ − y_1_y_2_.

The expected effect in Loewe model is the effect that would be achieved if a drug was combined with itself: y_e_ = y_1_(x_1_ + x_2_) = y_2_(x_1_ + x_2_), where x_1_ and x_2_ are doses of individual compounds.

The expected effect in ZIP model is the effect that would be achieved if the two drugs do not potentiate each other, i.e., both the assumptions of the Loewe model and the Bliss model are met [4].

Depending on the differences y_o_–y_e_ between the observed (y_o_) and expected (y_e_) effects, a drug combination can be classified as synergistic, antagonistic, or non-interactive [5]. If difference is positive (negative) drug combination exhibits synergistic (antagonistic) effect. If difference is near to zero, drug combination is non-interactive. The differences y_o_–y_e_ were usually used as drug synergy scores in various computational studies (see below).

Various software is currently available to calculate the synergistic effects of compound combinations using these models [6], including Combenefit [7], SynergyFinder [4] and Synergy [8].

Over the past decades, freely available databases with anti-cancer drug combinations with information of dose-response curves and synergy values based on above-mentioned models have been created [6]. Such databases as AstraZeneca-Sanger DREAM [9], Merck & Co. [10], NCI-ALMANAC [11], SYNERGxDB [12], or DrugCombDB [13] contain descriptions of cytotoxic activity based on the series of conducted experiments. The NCI-ALMANAC is a largest database of anti-neoplastic agent combinations that was published by the US National Cancer Institute (NCI) in 2017. The data was obtained by high-throughput screenings and include 304,549 combination experiments of 104 investigational and approved drugs across 60 cancer cell lines forming the NCI-60 panel. The synergy level is quantified by the ComboScore, a modified version of the Bliss model [11].

Despite existence of various databases of synergistic anti-neoplastic drug combinations, they cannot contain all possible combinations of anti-cancer and other drugs and drug-like compounds. Moreover, identification of successful drug combinations through experimental exploration is a time-consuming and expensive task due to the size of the combination space. One of the significant contributors to speed up traditional approaches is an extensive range of computational methods. In recent years several computational models were built to predict drug synergy. They are usually based on machine learning including development of deep learning architectures and various compound-based and cell-line-based features [5,14,15,16,17,18,19,20,21,22,23,24,25,26,27,28]. Compound-based features include known drug targets and drug-induced gene expression profiles in cell lines, cell-line-based features include various OMICs data profiles, e.g., gene transcription profile in a particular cell line.

For instance, Xia and co-authors [27] extracted drug pair response data for 54 drugs from NCI-ALMANAC against 59 cancer cell lines. Drug descriptors were computed with the Dragon software package based on the 2D structure of selected drugs. Cell line molecular features for selected cell lines were represented by three types of assays: gene expression levels, microRNA expression levels, and proteomics data. Drugs descriptors and cell line molecular features were used as input data for a deep neural network. The output of neural network was modified ComboScore. The performance of predictive models was measured with 5-fold cross-validation. The coefficient of determination for the best model was 0.944.

Julkunen and co-authors [28] developed a computational model for predicting drug combination responses of cancer cell lines based on the NCI-ALMANAC data. They randomly selected 50 drugs, for which drug pair response data against all 60 cancer cell lines was extracted. Chemical descriptors of selected drugs were generated using the rcdk R package. Gene expression profiles for selected cell lines were obtained from the rcellminer R package. The authors chose 0.5% of the genes with the highest variance across cell lines as genomic descriptors. Genomic and chemical descriptors were integrated into the prediction model utilizing a higher-order factorization machine. The output of the model was predicted ComboScore value. The model parameters (rank and regularization parameters) were optimized, and the performance of the best model was measured with the nested cross-validation procedure (10 outer folds CV, 5 inner folds CV). The Pearson and Spearman correlation for predicting new drug combinations achieved the values of 0.95 and 0.88, respectively.

Preuer and co-authors [23] developed a deep learning-based approach—DeepSynergy, that uses chemical and genomic information as features to learning and Merck & Co. synergy data [10]. They considered synergy pairs as having Loewe score more than 30 that correspond to top 10% pairs with the highest scores. Cross-validation procedure demonstrated classification AUC value of 0.90.

Yan and co-authors developed H-RACS tool [26] which is based on AstraZeneca [9] and Merck & Co data [10] (1380 drug combinations and 116 cell lines) and seven machine learning methods. They used various cell line-based features and drug-based features which included chemical descriptors and features of drug targets in protein-protein interaction network. Synergy pairs were considered as having Loewe score more than 30. H-RACS showed strong extendibility with AUC of 0.84. H-RACS is available as a free web application which requires smiles representation of molecule and UniProt identifiers of its known protein targets as input [26].

In spite of the high value of accuracy demonstrated by the above-mentioned studies, they have some essential drawbacks that hinder their application potential. First, they usually require a large number of compound-based features, e.g., drug-induced transcription profiles, drug-target interactions etc. Since the corresponding data absent for new compounds, the methods can be only applied to well-studied drugs. Second, these studies used randomly created sets for cross-validation, which can be considered as insufficiently rigorous validation procedure for the evaluation of drug-drug interactions because one or both drugs from the tested pair of drugs may stay in pairs from the training set [29]. This situation may lead to self-recognition problem and prediction accuracy values may be overestimated. Third, in most cases the developed models exist in form of source code and cannot be applied by a wide range of researchers.

Earlier, we developed the CLC-Pred web application to predict the cytotoxicity of compounds against hundreds of human tumor and non-tumor cell lines using only their structural formulas [30,31]. In the present study, we have developed a web-application that predicts the synergistic anti-neoplastic effect of compound combinations based on structure-activity relationship (SAR) analysis. The built SAR models were based on NCI-ALMANAC experimental data, and the Pairs of Substances Multilevel Neighbourhoods of Atoms (PoSMNA) descriptors that were developed to describe drug-drug interactions rather than single compounds, and modified naive Bayes approach that was earlier developed to deal with highly imbalanced datasets [32,33,34]. To estimate the accuracy of the predictions, we performed stricter “compounds out” validation procedure [29]. This included checking that the pairs of compounds in the independent test sets did not contain any individual compounds that were already present in the pairs from the training set.

The best created SAR models were incorporated in a freely available web application, CLC-Pred Synergy (https://way2drug.com/clc-pred-syn/, accessed on 4 June 2026), which predicts the cytotoxic synergy effect of drug pairs in NCI60 cell lines. It requires only the structural formulas of compounds and can be used to analyze new, even unsynthesized molecules.

## 2. Results

### 2.1. Main Results

The analysis of the NCI-ALMANAC database has enabled us to extract cytotoxic activity data for 99 drugs (out of 104) that are suitable for SAR modeling by PASS DDI, as well as 4851 pairs of drugs against NCI60 cancer cell lines. The extracted data points were divided into 1500 datasets, corresponding to all combination of cancer cell lines, synergy models and thresholds (see Material and Methods).

**Figure 1:**
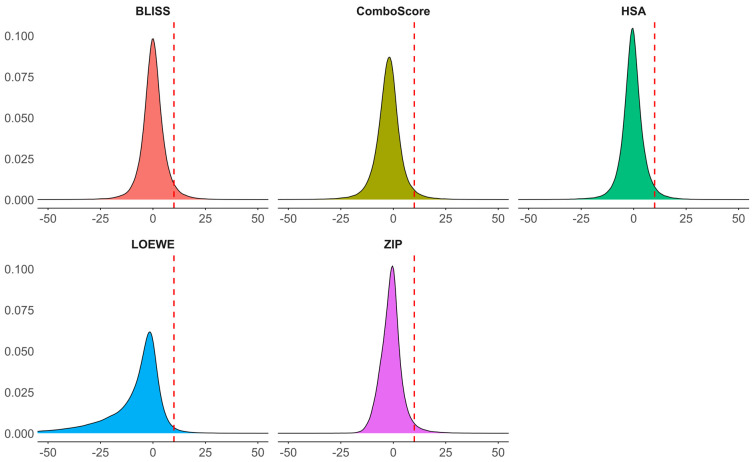
The distribution of Bliss, ComboScore, HSA, Loewe, and ZIP values. The dotted red line represents the established threshold of 10. A total of 264,953 synergy scores for combinations such as drug-drug-cell line were used to create each distribution.

In our study, we used Bliss, ComboScore, HSA, Loewe, and ZIP values to create SAR models. These values represented the difference between expected and observed growth fraction in a cell line according to the Bliss, ComboScore, HSA, Loewe, and ZIP synergy models (see Introduction). The positive values correspond to the synergistic combination, while the negative values indicate the antagonistic interaction. The higher the values, the stronger the observed cytotoxic synergism. Figure 1 shows the distributions of these values. They exhibit distinct shapes and variances: Loewe shows a left-skewed pattern, whereas ComboScore, HSA, Bliss, and ZIP exhibit sharper, more symmetric peaks centered near zero.

**Figure 2:**
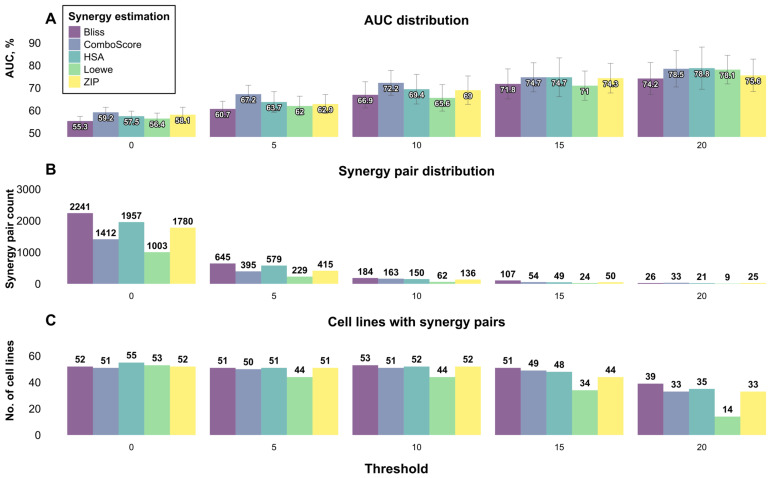
(**A**) The mean AUC values obtained for Bliss, ComboScore, HSA, Loewe and ZIP synergy models with different thresholds; (**B**) The number of synergy pairs obtained using Bliss, ComboScore, HSA, Loewe and ZIP synergy models with different thresholds; (**C**) The number of created SAR models with non-zero synergistic drug pairs in the datasets.

Synergy values from 0 to 30 were used as thresholds to create classification models in previous studies [5,15,19,20,21,22,23,24,25]. To investigate the number of synergistic drug pairs and prediction accuracies, Bliss, ComboScore, HSA, Loewe, and ZIP values from 0 to 20 with a step of 5 were considered as thresholds. We did not use thresholds of 25 and 30 because too few synergistic drug pairs remained. According to the thresholds all drug combinations in each dataset were divided into 2 classes: “synergistic” and “non-synergistic”. As a result, we created 1500 datasets and 1500 appropriate SAR models by PASS DDI software (see Materials and Methods). The performance of models’ prediction (AUC) was evaluated by the LOO CO CV (Figure 2).

The mean AUC values obtained by LOO CO CV increase with increasing synergy thresholds (Figure 2A); however, the mean number of synergistic drug pairs (Figure 2B) and number of cell lines with non-zero synergistic drug pairs decrease (Figure 2C). A threshold of Bliss, ComboScore, HSA, Loewe and ZIP was established equals to 10 for further research to balance between accuracy of prediction and number of synergistic pairs in datasets. A synergy score of 10 means that the observed drug combination effect was 10% greater than the effect expected from the null model (Bliss, ComboScore, HAS, Loewe or ZIP).

**Figure 3:**
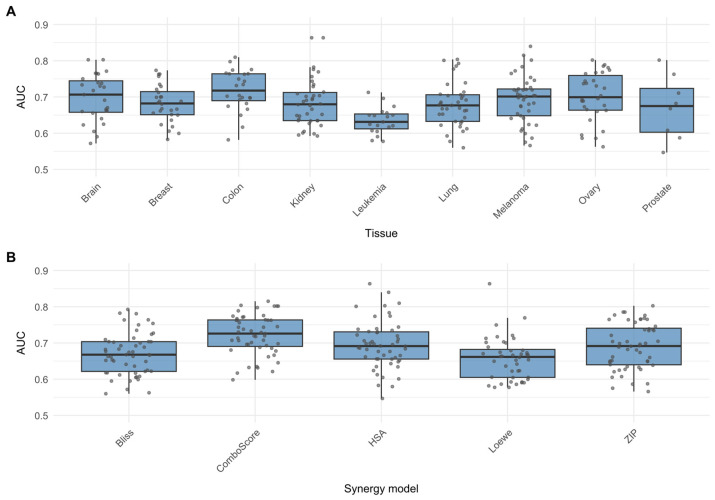
The comparison of distribution of AUC values between (**A**) tissues of tumor origin; (**B**) synergy models.

After selection the models with threshold of 10, we compared AUC values between tissues of tumor origin (Figure 3A) and synergy models (Figure 3B). Figure 3A demonstrates that the best AUC was obtained for colon cancer cell lines (mean AUC = 0.718), and the worst for leukemia cell lines (mean AUC = 0.635). Figure 3B shows that the use of ComboScore synergy model provides the highest accuracy (mean AUC = 0.722), whereas the use of Loewe model associated with the worst accuracy (mean AUC = 0.656).

**Figure 4:**
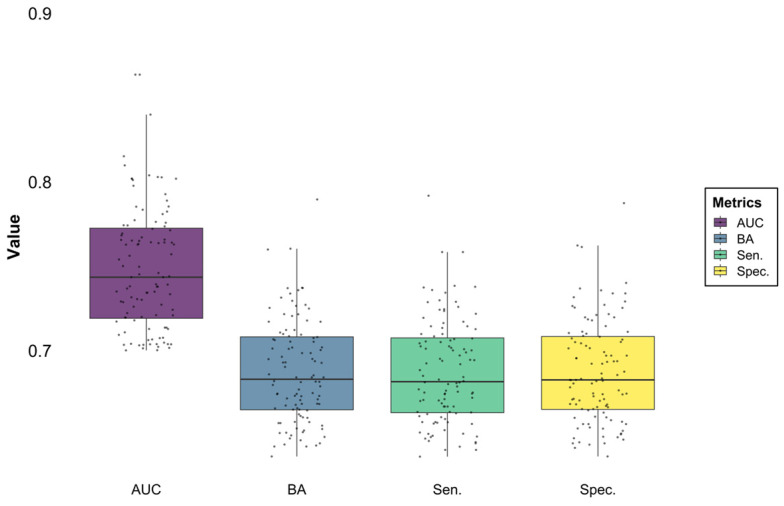
The distribution of AUC, sensitivity (Sen), specificity (Spec) and balanced accuracy (BA) values for selected 104 SAR models.

Finally, we selected only models with AUC values exceed 0.70 at LOO CO CV procedure (Figure 4). As a result, we obtained 104 SAR models corresponding to 45 cell lines with mean AUC of 0.749 (90% CI: 0.700–0.790), sensitivity of 0.687, specificity of 0.687, balanced accuracy of 0.687, and average number of synergy pairs of 114 (see Table S1).

### 2.2. CLC-Pred Synergy Web Application

The above mentioned 104 SAR models with a sufficient performance prediction were selected to create a freely available web application CLC-Pred Synergy for predicting the probability of a drug pair to reveal the synergistic cytotoxicity against 45 NCI60 cell lines (https://www.way2drug.com/clc-pred-syn/, accessed on 4 June 2026).

To obtain a prediction, users have to input structural formulas for both compounds in the combination. The interface provides the opportunity to search for the structure of known drugs using their names, or to enter their SMILES. Only two structural formulas are required for CLC-Pred Synergy, which we considered a significant advantage. This allows us to estimate the potential synergy in antineoplastic effect, even for make-on-demand compounds during the earliest stages of drug development.

**Figure 5:**
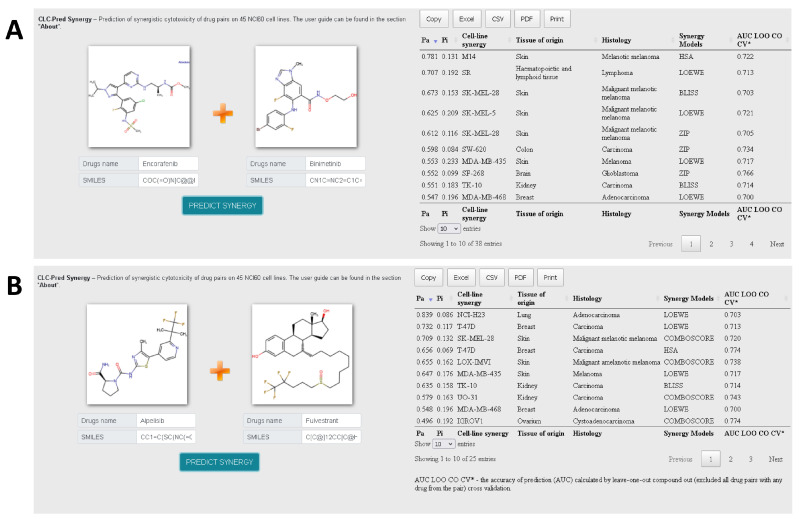
Prediction of cytotoxic synergy for the combination of (**A**) encorafenib and binimetinib; (**B**) alpelisib and fulvestrant at CLC-Pred Synergy.

The output of the web application CLC-Pred Synergy is the prediction of the synergistic cytotoxicity effect for two specified drugs against 45 cancer cell lines (Figure 5). The result is provided in the form of a table with 7 columns, where each row describes the prediction for one cell line using one of the 5 synergy models (Bliss, ComboScore, HSA, Loewe, or ZIP). The column “Pa” contains the probability that a drug pair will belong to the “synergistic” class. The column “Pi” contains the probability that a drug pair will belong to the “non-synergistic” class. The values in both columns range from 0 to 1. A drug pair with the highest Pa–Pi value has the best chance to reveal synergism in a further in vitro experiment [35]. The predictions are sorted by decreasing Pa–Pi value, so that the most confident predictions are at the top of the table (see Figure 5).

The Pa and Pi values can be used to estimate the applicability domain for a new drug pair. Probability Pa (Pi) reflects the similarity of a molecule pair under prediction with the structures of molecule pairs that are the most typical in a subset of “synergistic” (“non-synergistic”) pairs in the training set. If both Pa and Pi are low and the Pa–Pi value is near zero, then the drug pair is not similar to any subset of drug pairs in the training set. Thus, the prediction of being synergistic or non-synergistic cannot be performed for the pair (see more details in Supplementary Materials). The corresponding predictions are at the bottom of the sorted prediction list (see Figure 5).

The columns “Cell-line synergy”, “Tissue of origin”, and “Histology” characterize the cancer cell line and the panel to which it belongs. The column “AUC LOO CO CV” shows the accuracy of the prediction obtained during the validation phase. The result can be downloaded in CSV, Excel, or PDF formats.

Figure 5A shows an example of cytotoxic synergy prediction for the combination of encorafenib and binimetinib by CLC-Pred Synergy. Such a combination was approved by the FDA for the treatment of melanoma in 2018 [36,37]. Although these drugs were not in the training sets of SAR models, CLC-Pred Synergy predicted 5 skin cell-lines (see Table 1), including M14 (melanoma cell line) with the Pa > 0.7. Interestingly that this combination is specifically used to treat unresectable or metastatic melanoma which harbor a BRAF V600E or V600K mutation. M14 cell line also has this mutation. Synergy effect for other melanoma cell lines (SK-MEL-28, SK-MEL-5) with different synergistic scores were also predicted with Pa > 0.5. Interestingly, that synergistic effect was also predicted for MDA-MB-435, which was earlier considered as breast cancer cell line, but it was later found that it was contaminated by melanoma cells and therefore MDA-MB-435 reveals properties of melanoma [38]. This may serve as an additional positive criterion that this drug combination may have a synergistic effect against melanoma cells. Another example is combination of PIK3CA inhibitor alpelisib with selective estrogen receptor degradator fulvestrant. The combination was approved for the treatment of hormone receptor-positive (HR+), HER2-negative advanced or metastatic breast cancer in patients with a PIK3CA gene mutation [39,40]. The highest Pa-Pi equals 0.615 was obtained for T-47D breast cancer cell line which also harbors this mutation (Figure 5B). The synergistic effect for NCI-H23 (lung adenocarcinoma) and SK-MEL-28 (melanoma cell line) was also predicted for this combination with high Pa values (more 0.7). It may be the reason for experimental study of this combination for the treatment of lung cancer and melanoma.

### 2.3. Validation of CLC-Pred Synergy on Drug Combinations Used in Clinics

We performed a literature search and found 23 synergistic drug pairs that are currently approved to treat glioblastoma, melanoma, breast, colon, kidney, lung, and ovarian cancers (Table 1, detailed information on drug combinations presented in Table S2). None of the 23 synergistic drug pairs were presented in the training sets. We found that 17 out of 23 drug pairs (73.9%) were predicted to be synergistic for the appropriate cell lines (95% CI: 0.5652–0.9130). Importantly, that 10 of 23 drug pairs did not contain both individual drugs in pairs from trainings sets, and 8 of 10 pairs (80%; 95% CI: 0.5–1.0) were predicted to be synergistic (see Table 1).

## 3. Discussion

In our study, we analyzed NCI-ALMANAC data and extracted data about the cytotoxicity effect of 99 FDA-approved drugs and their pairwise combinations against 60 cancer cell lines. The extracted data points were divided into 1500 datasets, one dataset for each cancer cell line, synergy model and threshold. We used five synergy models: Bliss, ComboScore, HSA, Loewe, ZIP. Thresholds on synergy values were established from 0 to 20 with a step of 5. According to the thresholds all drug combinations in each dataset were divided into 2 classes: “synergistic” and “non-synergistic”. Based on this data and the structures of selected drugs, we created SAR models using the PASS DDI software. The performance prediction for the created SAR models was estimated by LOO CO CV procedures.

We found that the threshold of 10 for all synergy models (Bliss, ComboScore, HSA, Loewe, and ZIP) allowed to obtain the best result: the relatively high number of synergistic pairs in the datasets and enough accuracy of prediction (see Figure 2). A synergy score of 10 means that the observed drug combination effect was 10% greater than the effect expected from the null synergy model. With this threshold, we obtained 104 SAR models corresponding to 45 cell lines with the mean AUC value of 0.749 (90% CI: 0.700–0.790), sensitivity, specificity and balanced accuracy values of 0.687, minimal AUC of 0.70, and average number of synergy pairs of 114. Synergy values from 0 to 30 were used as thresholds to create classification models in previous studies [5,15,19,20,21,22,23,24,25]. Abd El-Hafeez with colleagues also used threshold of 10 to divide drug pairs into synergistic and non-synergistic [5]. Moreover, the threshold of 10 is also recommended in widely applied SynergyFinder [4] software. The recommendation to use a threshold of 10 in SynergyFinder is a practical, empirically derived rule designed to help researchers interpret synergy scores consistently across studies. Synergy scores from 0 to 10 are often associated more with technical noise than with real drug synergy. Thus, a cutoff of 10 provides a practical “buffer zone” of low confidence. Scores within this range (from 0 to 10) are considered additive, meaning there is not enough evidence to reliably call the interaction synergistic. By using a threshold of 10, weak or borderline results are filtered out, and combinations with a clear and substantial deviation from additivity are selected. Consequently, the selected threshold of 10 is in accordance with generally accepted practice and allows researchers to select drug pairs with strong synergy.

A limitation of this approach is the inherent trade-off between prediction accuracy and the number of synergistic drug pairs. Thus, choosing an optimal threshold reduced the number of cell lines for which predictions could be made.

Previous studies [5,15,19,20,21,22,23,24,25] used random cross-validation to estimate the accuracy of synergy prediction. Moreover, in most previous studies, triplets such as drug–drug–cell line were randomly divided into training and test sets. This situation may lead to a self-recognition problem, and prediction accuracy values may be overestimated. Since rigorous validation is extremely important for methods that can be applied to solve various medical problems, including screening tools or biological activity prediction tools [41], we calculated accuracy values using the LOO CO CV procedure. The procedure is related to the “compounds out” validation strategy when all pairs of drugs with any drug from the test set are excluded from the training set before the prediction of synergy for drug pairs in the test set [29]. The applied validation procedure reflects a situation where both compounds in a pair are new and absent from the pairs in the training set. This explains why the observed prediction accuracy (AUC of 0.749) was only “moderate” and lower than that in previous studies (AUC > 0.80) [15,19,20,21,22,23,24]. Some researchers performed a Leave Drugs Out procedure, which is similar to traditional random cross-validation. In those studies, drug pairs were randomly divided into training and test sets across all cell lines [23,26]. For example, DeepSynergy [23], a deep learning-based approach, uses chemical and genomic information as features for learning and the Merck & Co. synergy data [10]. Synergy pairs were considered as having a Loewe score greater than 30. The Leave Drugs Out cross-validation procedure showed an AUC value of 0.90. The H-RACS approach [26] is based on AstraZeneca [9] and Merck & Co. data [10], various cell line-based and drug-based features, and employs seven machine learning methods. Synergy pairs were also considered as having a Loewe score > 30. H-RACS demonstrated an AUC of 0.84 using the Leave Drugs Out cross-validation procedure. To compare our CLC-Pred Synergy approach with these previous studies, we calculated the AUC using the traditional leave-one-out cross-validation procedure, where each drug pair in each of the 104 datasets was subsequently excluded, and the remaining pairs were used to retrain the model. The average AUC was 0.84 (see Table S1 for details), which is comparable to the accuracy of DeepSynergy, H-RACS, and other approaches.

To increase the accuracy of prediction, novel structure-based feature generation methods can be used in the future, including calculation of structure embeddings using network-based methods. For instance, NEDD approach, which is based on the calculation of meta paths of different lengths in heterogeneous network of drug-drug, drug-disease and disease-disease associations, can be used to create the low dimensional representation vectors of drugs [42].

The approach was validated on 23 synergistic drug pairs that are currently approved to treat glioblastoma, melanoma, breast, colon, kidney, lung, and ovarian cancers (see Table 1). None of 23 synergistic drug pairs were presented in training sets. It was found that 17 out of 23 drug pairs (73.9%) were predicted to be synergistic for the appropriate cell lines (95% CI: 0.5652–0.9130). Importantly, 10 of the 23 drug pairs did not contain both individual drugs in pairs from the training sets, and 8 of these 10 pairs (80%; 95% CI: 0.5–1.0) were predicted to be synergistic (see Table 1). However, it should be noted that the number of approved synergistic drug pairs is small and the confidence intervals for the percentages of correctly predicted pairs are wide. This hinders the validation results. Nevertheless, the obtained accuracies are comparable with the accuracies (AUC of 0.749) calculated by the LOO CO CV procedure on large datasets, which suggests the acceptable prediction capacity of the created models.

Six out of 23 synergistic pairs were incorrectly predicted as non-synergistic. Abemaciclib–anastrozole combination used to treat breast cancer seems to be predicted incorrectly. It is more similar to non-synergistic pairs of the training set (Pi = 0.8) than to synergistic pairs (Pa = 0.027, Pa–Pi = −0.773). The other five pairs have low values of both Pa and Pi (see Table 1). This can be interpreted as the drug pair not being similar to either synergistic or non-synergistic pairs in the training set. For example, the palbociclib–letrozole combination used to treat breast cancer has Pa = 0.239 and Pi = 0.335 (Pa–Pi = −0.096). Thus, these drug pairs may be considered as out of the applicability domain of the developed models and cannot be predicted by CLC-Pred Synergy.

The observed results demonstrate the applicability of our approach to predict synergistic combinations of compounds with anti-neoplastic effects based on their structural formulas. One hundred and four models with the highest accuracy of prediction were used for the creation of a publicly available CLC-Pred Synergy web application (https://www.way2drug.com/clc-pred-syn/, accessed on 4 June 2026). It provides the opportunity to predict the probability of a drug pair to reveal synergistic cytotoxicity against 45 cancer cell lines. In contrast to methods of other authors, it requires only the structural formulas of drugs as the input.

Figure 5 and Table 1 show that the prediction results for the same cell line contain outputs for several synergy models (Bliss, ComboScore, HSA, Loewe, ZIP). Creating an ensemble of the five synergy models might potentially improve prediction accuracy. However, it has been shown that synergy scores calculated by Bliss, HSA, Loewe, and ZIP are weakly correlated [43]. Therefore, we created separate models, leaving users to choose the appropriate ones. Nevertheless, the more synergy models included in the prediction output, the higher the probability that the drug pair will be synergistic for the cell line.

The limitation of CLC-Pred Synergy is its applicability to only small organic molecules, whereas monoclonal antibodies are often used in oncology, including their combinations with small molecule drugs. Other types of therapeutics, such as antibody-drug conjugates or peptide-based drugs, also cannot be used. Earlier, we demonstrated the applicability of MNA descriptors for predicting properties of peptides and proteins [44,45,46,47,48]. Unfortunately, we cannot currently create corresponding models for synergy prediction because of the absence of such data for peptides and antibodies.

Another limitation is that the approach does not provide information on potential mechanisms of synergy. In future studies, potential synergy mechanisms could be estimated based on drug-target interaction prediction using structure-activity relationships coupled with gene-disease ranking in multiplex networks [49,50,51,52]. Another promising approach is network pharmacology, which integrates the analysis of molecular networks and omics data. This analysis, combined with synergy prediction by CLC-Pred Synergy or other machine-learning-based approaches, could provide a deeper understanding of the antineoplastic potential of drug combinations [53,54].

## 4. Materials and Methods

### 4.1. NCI-ALMANAC Database

Information about the synergistic anti-neoplastic activity of drug pairs was obtained from the NCI-ALMANAC (A Large Matrix of Anti-Neoplastic Agent Combinations) database [11]. NCI-ALMANAC provides the experimental data for over 5000 pairs of FDA-approved anti-cancer agents against a panel of 60 well-characterized human tumor cell lines (NCI60 screen) representing 9 tissue types [55]. The important advantage of data in NCI-ALMANAC is the standard testing protocol, which has formed the basis for more than 300,000 assays (https://dtp.cancer.gov/discovery_development/nci-60/methodology.htm, accessed on 5 May 2026). The cytotoxic effects of each drug out of a total of 104 were measured, both individually and in pairwise combinations. The revealed difference in the growth fraction for a cell line exposed to two drugs separately and in a drug-drug pair was used to compute “ComboScore” by utilizing a modification of Bliss independence [11,56]. The information on pairs of compounds, which contains nearly 3 million data points, including ComboScore, was obtained from the NCI ALMANAC Study Results (https://dtp.cancer.gov/ncialmanac, accessed on 5 May 2026). The information on Bliss, HSA, Loewe, and ZIP precomputed values, obtained using NCI-ALMANAC dose-response data, was downloaded from DrugCombDB [13] (http://drugcombdb.denglab.org/main, accessed on 5 May 2026). The original NCI-ALMANAC data contain 9 or 15 (3 × 3 or 5 × 3 concentration matrices) data points for each of the drug pairs with different doses of combination components. Whole concentration matrices are required to calculate Loewe and ZIP values, whereas Bliss, ComboScore, and HSA values are calculated for each dose combination. Therefore, mean values of Bliss, ComboScore, and HSA scores were calculated across all doses combinations to obtain single value for each pair of compounds. As a result, each drug-drug pair was associated with Bliss, ComboScore, HSA, Loewe, and ZIP values which represented the difference between expected and observed growth fraction in a cell line (see Introduction). The positive value corresponds to the synergistic combination, while the negative value indicates the antagonistic interaction. The higher the values, the stronger the observed cytotoxic synergism.

### 4.2. Training Sets

NCI-ALMANAC was used as a source of synergistic and non-synergistic pairs to create training sets. Drug-drug pairs were annotated as “synergistic” or “non-synergistic” based on the different thresholds of Bliss, ComboScore, HSA, Loewe, and ZIP values: 0, 5, 10, 15, and 20. The corresponding threshold values were chosen according to previous studies [5,15,19,20,21,22,23,24,25]. From one dataset, we derived 1500 training sets by taking all combinations of 60 cancer cell lines, 5 thresholds, and 5 synergy models: 60 × 5 × 5 = 1500.

The structural formulas of drugs were download in SDF (Structure-Data file) format [57] from the NCI ALMANAC Study Results (https://dtp.cancer.gov/ncialmanac, accessed on 5 May 2026).

As a result, 1500 SD files were created, where drug pairs are related with their chemical structures and the class (“synergistic” or “non-synergistic”).

### 4.3. SAR Models

**Figure 6:**
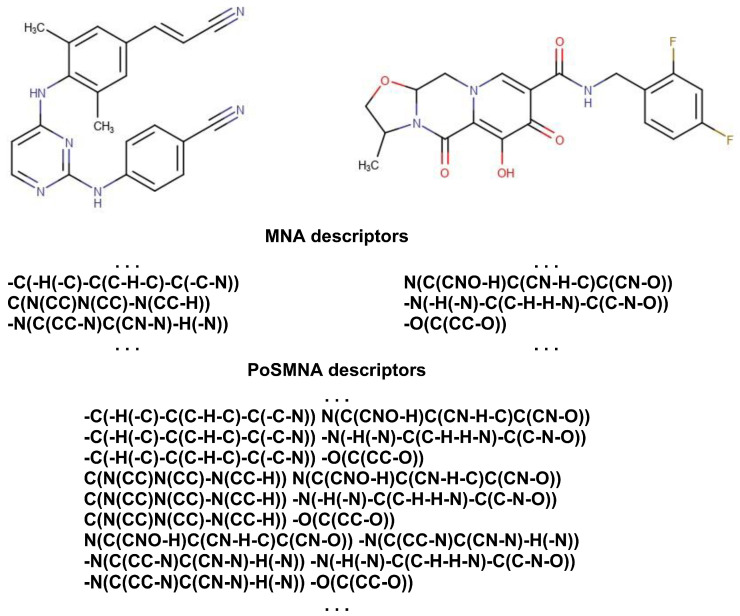
Representation of the pair of molecules by the Pairs of Substances Multilevel Neighborhoods of Atoms (PoSMNA) descriptors.

The original PASS DDI software (version 1.0) was used to create SAR models for predicting the synergistic cytotoxic effect in relation to the corresponding cell line based on the prepared training sets [32,33,58]. This is a version of the PASS (Prediction of Activity Spectra for Substances) software, which provides prediction of effects for pairs of compounds (DDI—drug-drug interaction). PASS uses the MNA (Multilevel Neighborhoods of Atoms) descriptors to represent the structure of molecules [59]. The Pairs of Substances MNA (PoSMNA) descriptors have been implemented in the PASS DDI software for predicting DDI by describing a pairwise combination of molecules instead of a single molecule. A set of PoSMNA descriptors is the product of sets of second-level MNA descriptors of two molecules in a pair: the first descriptor of the first molecule is linking (in lexicographic order) together with each descriptor of the second molecule, etc. As a result, each of the pairs of structures is represented as a set of PoSMNA descriptors (see Figure 6) [32,33,58]. SD files with structural formulas and class labels were used as the input for the PASS DDI software. Structures of 99 out of 104 compounds were suitable for SAR modeling by PASS DDI, since they were small-molecule organic compounds (molecular weight less than 1500 daltons) and contained more than three carbon atoms. The five excluded drugs include three inorganic compounds (arsenic trioxide, cisplatin, and hydroxyurea) and two compounds with high molecular weight (bleomycin sulfate and dactinomycin). PASS DDI created SAR models correlating the sets of PoSMNA descriptors with the synergy class using a modified naive Bayes classifier [34]. As an output, 1500 SAR models were created across 60 cancer cell lines, 5 types of synergy values and 5 thresholds.

PASS DDI calculates two estimates of probabilities for a new pair of compounds: Pa is the probability that the combination will be synergistic, and Pi is the probability that it will not be synergistic for the appropriate cell line. The greater the difference between Pa and Pi, the greater the likelihood of obtaining synergy in an experiment [34].

### 4.4. Model Validation

**Figure 7:**
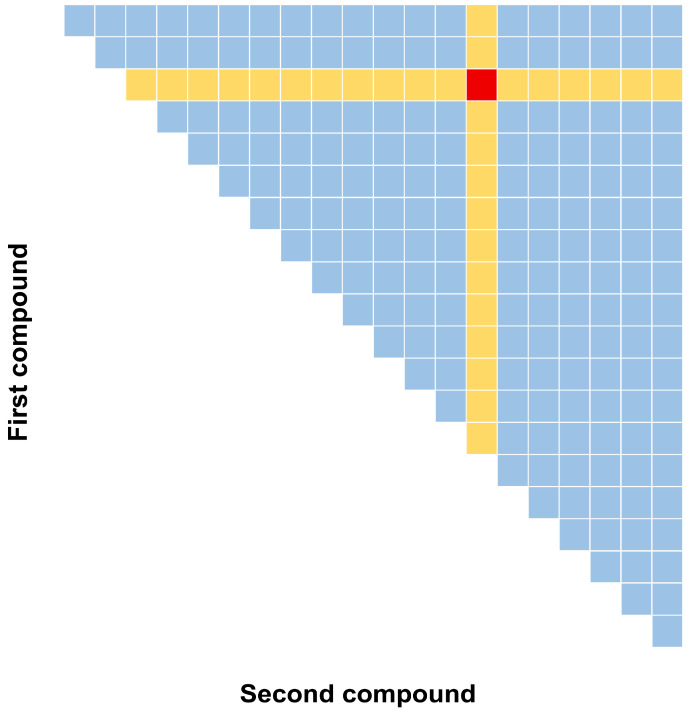
The main idea behind the “compounds out” cross-validation procedure. The rows and columns are individual compounds; the cells are pairs of compounds. The blue color represents pairs in the training set; the red color represents the testing pair; the yellow color represents excluded pairs.

The accuracy of the models was estimated using “compounds out” cross-validation procedure (Figure 7). The “compounds out” validation strategy means that all pairs of drugs with any drug in the test set are excluded from the training set before the prediction of synergy for drug pairs in the test set [29]. Due to the removal of several pairs from the calculations (as shown in Figure 7) and the significant decrease in the number of pairs in the training set, PASS DDI implemented leave-one-out compounds-out cross-validation (LOO CO CV) instead of k-fold cross-validation. PASS DDI calculates various accuracy estimates for SAR models, including AUC (Area Under the Receiver Operating Characteristic Curve), sensitivity, specificity, and balanced accuracy, using LOO CO CV.

95% confidence intervals for accuracy values were calculated by bootstrap analysis.

## Figures and Tables

**Table 1:** Synergistic drug pairs that are currently approved to treat glioblastoma, melanoma, breast, colon, kidney, lung, and ovarian cancers, and are not presented in training sets from NCI-ALMANAC. Table contains only one predicted cell line per synergistic pair with maximal PASS DDI Pa-Pi value. Pa-Pi is a maximal of Pa-Pi values calculated for several synergy models. Asterisk (*) means drug pairs that do not contain both individual drugs in pairs from training sets. (-) means that the synergistic effect was not predicted for any of cell lines.

Combination	Cancer Type	Number of Predicted Cell Lines	Pa-Pi	Most Probable Cell Line	Synergy Models
Dabrafenib–Trametinib *	Brain cancer (glioblastoma)	-	−0.071	-	-
Abemaciclib–Anastrozole	Breast cancer	-	−0.773	-	-
Abemaciclib–Fulvestrant	Breast cancer	-	−0.170	-	-
Abemaciclib–Tamoxifen	Breast cancer	-	−0.335	-	-
Alpelisib–Fulvestrant	Breast cancer	3	0.615	T-47D	HSA, Loewe
Capivasertib–Fulvestrant	Breast cancer	5	0.853	T-47D	HSA, Loewe
Neratinib–Capecitabine	Breast cancer	3	0.211	MDA-MB-468	Bliss, Loewe, ZIP
Palbociclib–Fulvestrant	Breast cancer	5	0.440	T-47D	HSA, Loewe
Palbociclib–Letrozole	Breast cancer	-	−0.096	-	-
Ribociclib–Letrozole	Breast cancer	1	0.150	BT-549	HSA
Ribociclib–Fulvestrant	Breast cancer	3	0.290	T-47D	HSA, Loewe
Encorafenib–Binimetinib *	Colorectal cancer	3	0.514	SW-620	Bliss, HSA, ZIP
Belzutifan–Cabozantinib *	Renal cancer	3	0.448	TK-10	Bliss, HSA
Belzutifan–Lenvatinib *	Renal cancer	3	0.490	TK-10	Bliss, HSA
Lenvatinib–Everolimus	Renal cancer	4	0.519	TK-10	HSA, Loewe, ZIP
Dabrafenib–Trametinib *	Lung cancer	-	−0.090	-	-
Encorafenib–Binimetinib *	Lung cancer	1	0.071	NCI-H23	Bliss, HSA
Avutometinib–Defactinib *	Ovarian cancer	2	0.162	OVCAR-5	ComboScore, ZIP
Olaparib–Cediranib *	Ovarian cancer	4	0.551	IGROV1	ComboScore
Paclitaxel–Relacorilant	Ovarian cancer	6	0.970	OVCAR-5	ComboScore, ZIP
Dabrafenib–Trametinib *	Melanoma	4	0.416	M14	HSA
Encorafenib–Binimetinib *	Melanoma	5	0.650	M14	HSA
Vemurafenib–Cobimetinib	Melanoma	6	0.916	SK-MEL-5	ComboScore, HSA, Loewe, ZIP

## Data Availability

Experimental data on the existing synergistic and non-synergistic drug pairs, and SD file with structural formulas were obtained from a publicly available NCI-ALMANAC database, (https://dtp.cancer.gov/ncialmanac, accessed on 5 May 2026). The information used for SAR modeling, including synergy values and filtered 99 structures in SD file, is also available at section “About” of CLC-Pred Synergy web application. CLC-Pred Synergy is freely available at https://www.way2drug.com/clc-pred-syn/ (accessed on 4 June 2026).

## Supplementary Materials

The following supporting information can be downloaded at: https://www.mdpi.com/article/10.3390/ijms27125208/s1. References [60,61,62,63,64,65,66] are cited in the supplementary materials.

## Abbreviations

The following abbreviations are used in this manuscript:

CLC	Cell Line Cytotoxicity
DDI	Drug-Drug Interaction
LOO CO CV	Leave-One-Out Compounds Out Cross-Validation
MNA	Multilevel Neighborhoods of Atoms
PASS	Prediction of Activity Spectra for Substance
PoSMNA	Pairs of Substances MNA
SAR	Structure-Activity Relationship
SDF	Structure-Data File
