# Supplementary material for: CLC-Pred Synergy: Web Application for Predicting Pairwise Drug Combinations with Synergistic Activity Against NCI60 Cancer Cell Lines
Source: Int J Mol Sci. 2026 Jun 9;27(12):5208. doi: 10.3390/ijms27125208 (PMC13299545; doi:10.3390/ijms27125208)
Supplement: Supplementary file 1 [file ijms-27-05208-s001.zip › Supplementary text.pdf]

Supplementary text

CLC-Pred Synergy: Web Application for Predicting Pairwise Drug Combinations with Synergistic Activity Against NCI60 Cancer Cell Lines

Vladislav S. Sukhachev, Sergey M. Ivanov, Anastasia V. Rudik, Arseny R. Dublin, Dmitry A. Filimonov, Alexey A. Lagunin, and Vladimir V. Poroikov

Analysis of structure-activity relationships and prediction of activity for new compounds using PASS approach

Biological activity of organic compound is the result of its interaction with biological object. It depends on the peculiarities of compound (molecular structure), biological object (species, sex, age, etc.), way of exposure (route of administration, dosage), biological assay peculiarities. Biological activity in PASS is described ***qualitatively*** ("active" or "inactive").

The **Biological activity spectrum** of an organic compound is the set of different kinds of biological activity that reflect the results of the compound's interaction with various biological entities. It represents the "intrinsic" property of a substance depending only on its structure. This superconcept goes in, we ensure opportunities for consolidation of big data from different source of information, since the information on described an organic compound from a single publication never cover all aspects of the biological action. In addition to that we follow the principle of "presumption of innocence": it is assumed in PASS that the compound does not have the biological activities that are not listed in its spectrum. Although we cannot exclude the situation when information on any activity of the organic compound was not found in the available sources or it possesses some biological activity, but the activity of this compound has not yet been tested. This approximation does not have an essential influence on results of analysis of "structure-activity" relationship and performed on this basis of the prediction due to statistical robustness of the PASS algorithm. It should be emphasized that arbitrary objective classification of organic compounds can be made the subject of prediction by PASS. If the appropriate classes are really determined by their structural features, the prediction of belonging to these classes can be quite successful. For instance, the range of certain quantitative value can be considered in PASS as "activity": if the value belongs to this range, then it is "active", and it is "inactive" in the other cases. It is clear that the applicability of PASS is broader than the prediction of biological activities.

Two-dimensional structural formula of an organic compound was chosen for the description of its **molecular structure**, because this is the only information about the compound available at the early stages of research (the compound can only be planned to synthesis). We have developed special molecular descriptors, which we call **MNA** descriptors (Multilevel Neighborhoods of Atoms), to describe the structure of a chemical compound. These descriptors were developed on the basis of approaches to the problem of "structure - property" relationships [1, 2]. MNA descriptors are based on the molecular structure representation, which includes the hydrogens according to the valences and partial charges of other atoms and does not specify the types of bonds: the nature does not know what is that "erased hydrogens" and the bonds order in many cases actually should be a fractional - for example, in an aromatic ring or in a -NO_2 fragment, - it is possible only to assert that whether there is rather stable chemical bond between the two atoms or not. In such form the structural formula becomes even formally unambiguous, and, for example, it does not depend on alternative methods of aromatic systems representation.

Based on described above molecular structure representation the MNA descriptors are generated for each of the atoms in a molecule as recursively defined sequence:

zero-level MNA descriptor for each atom is the mark *A* of the atom itself;

any next-level MNA descriptor for the atom is the sub-structure notation $A(D_1D_2...D_i...)$, where D_i is the previous-level MNA descriptor for i–th immediate neighbor's of the atom A. The neighbor descriptors $D_1D_2...D_i...$ are arranged in unique manner, e.g., in lexicographic order.

This iterative process can be continued to any level. It is important that the mark of atom may include not only the conventional atomic type symbol but also any additional information about the atom, e.g., that the atom is in a chain or some cyclic system or the atom is a site of biotransformation.

The molecular structure is represented in PASS by the set of unique MNA descriptors of the 1st and 2nd levels. In the second level MNA descriptors the atoms that does not belonged to any cycles are marked by «–».

An important feature of MNA descriptors is their openness: these descriptors are generated based on the structural formula itself, rather than based on any predefined list of structural fragments. Their other feature is that the structural integrity of fragments is conserved, in the sense that there is the corresponding fragment for each MNA descriptor.

The organic compounds are considered to be equivalent in PASS if they have the same set of MNA descriptors. Since MNA descriptors do not represent the stereochemical peculiarities of a molecule, the substances whose structures differ only stereochemically, are formally considered as equivalent.

The MNA descriptors are generated only if molecular structure corresponds to the following criteria:

- each of the atoms in a molecule must be presented by atom symbol from the periodic table. Symbols of unspecified atom A, Q, *, or R group labels are not allowed;
- each of the bonds in a molecule must be covalent bond presented by single, double or triple bond types only;
- molecular structure must include three or more carbon atoms;
- molecular structure must include only one component. Single atom parts like HCl, Cl-, OH-, Na+, etc., (hydrogen atoms do not take into account) are excluded from MNA descriptors generation;

A relevant error will be generated whenever a molecular structure does not correspond to these criteria or input data contains some other errors.

The general PASS **training set** includes about 1,500,000 thoroughly selected data records about structure and biological activity of organic compounds. The knowledgebase SAR Base is created in the process of training using PASS training set.

SAR Base includes a biological activities dictionary and MNA descriptors dictionary, data and knowledge on the "structure – biological activity" relationships, the database of structure of compounds from the training set with their biological activity spectra. The structure of the compound represented as a set of MNA descriptors. Unfortunately, it is currently impossible to make a large collection of biologically active compounds using only publicly available sources, for which assay results for all types of biological activity were known. For this reason, some types of biological activity in PASS training set represented by more than 10,000 of organic compounds, and others with just a few.

Different information sources include different terms for the same biological activity of organic compounds. Therefore, activity spectra in the PASS training set were standardized.

Algorithm of activity spectrum prediction description is based on well-known Bayesian approach. For the chemical compound C, which molecular structure is represented by the set $\{D_1, ..., D_m\}$ of m MNA descriptors, estimate the probability $P(A|C)$ that the compound C has an activity A. According to the Bayes formula:

$$P(A|C) = \frac{P(A)P(C|A)}{P(C)}$$

where $P(A)$ is the activity A *prior* probability; $P(C|A)$ is the conditional probability of compound C providing that it has activity A; $P(C)$ is the compound C prior probability.

On the assumption that descriptors $D_1, \ldots, D_m$ are independent one can write the probability $P(C|A)$ as the product of conditional probabilities for particular descriptors:

$$P(C|A) \cong P(D_1, \ldots, D_m|A) = \prod_{i=1}^{m} P(D_i|A)$$

This expression is approximately true since the MNA descriptors are a fortiori dependent due to their generation method. However, we have not acceptable alternatives, and we cannot forget about the approximation of the obtained formulas.

After simple transformations, we obtain the expression for the log-likelihood ratio of the conditional probabilities $P(A|C)$ for activity A and $P(B|C)$ for activity B as:

$$ln\left[\frac{P(A|C)}{P(B|C)}\right] \cong ln\left[\frac{P(A)}{P(B)}\right] + \sum_{i=1}^{m}\left\{ln\left[\frac{P(A|D_i)}{P(B|D_i)}\right] - ln\left[\frac{P(A)}{P(B)}\right]\right\}$$

In a particular case, where B is the lack of activity A, we obtain:

$$ln\left[\frac{P(A|C)}{1 - P(A|C)}\right] \cong ln\left[\frac{P(A)}{1 - P(A)}\right] + \sum_{i=1}^{m}\left\{ln\left[\frac{P(A|D_i)}{1 - P(A|D_i)}\right] - ln\left[\frac{P(A)}{1 - P(A)}\right]\right\}$$

The implication of the expression is quite clear: the logarithm of the posterior likelihood ratio is the sum of the logarithm of the a priori likelihood ratio and the sum of individual descriptors contributions. And, if the activity is not dependent on this descriptor, then $P(A|D_i) = P(A)$ and such descriptor has no affect on the results and its contribution to the sum is zero. This is the classical result of the probabilistic approach. But, apart from the already marked proximity, this result has another significant, well-known disadvantage: the contribution of some descriptors is too large and suppresses all other terms of the sum for which the conditional probability of activity is too close to 0 or 1, when they are present in the structure. The most pronounced effect could be in the situation, when for probabilities $P(A|D_i)$ we use the frequency estimates obtained based on analysis of the training set and the values 0 and 1 are the rule rather than the exception.

To overcome this problem one can try many different approaches and they were tested in the course of PASS development. The best result was obtained using so-called Fischer $ArcSin(2p - 1)$ conversion instead of $ln[p/(1 - p)]$: its shape coincides with the shape of $ln[p/(1 - p)]$ for almost all values of p, but $ArcSin(2 - 1)$ values are bounded by the values $\pm\pi/2$. The accuracy of prediction also improved after changing the sum of descriptor contributions by their average value, which apparently compensates for the assumption of descriptors independence. Logarithm of the a priori likelihood ratio contains a little information about specific predicted organic compound and can be omitted.

Bayesian approach described above explains why PASS prediction algorithm based on the following specific statistics: on the basis of a molecular structure represented by the set $\{D_1, \ldots, D_m\}$ of m MNA descriptors, the B_k values are calculated for each activity A_k:

$$B_k = \frac{S_k - S_{0k}}{1 - S_k \cdot S_{0k}}$$

$$S_k = Sin\left[\sum_i ArcSin(2P(A_k|D_i) - 1)/m\right]$$

$$S_{0k} = 2P(A_k) - 1$$

For each kind of activity, if for all descriptors of molecule $P(A_k|D_i) = 1$, then $B_k = 1$; if for all descriptors of molecule $P(A_k|D_i) = 0$, then $B_k = -1$; if the relationship between descriptors of molecule and activity A_k does not exist and $P(A_k|D_i) \approx P(A_k)$, then $B_k \approx 0$.

The PASS prediction algorithm uses the following data on the "structure-activity" relationships:

N is the total number of compounds in the SAR Base;

N_i is the number of compounds contained descriptor D_i in the structure description;

N_k is the number of compounds contained the activity A_k in the activity spectrum;

N_{ik} is the number of compounds contained both the activity A_k and the descriptor A_k.

The simplest frequency estimations of probabilities $P(A_k)$ и $P(A_k|D_i)$ are given by:

$$P(A_k) = \frac{N_k}{N}, \qquad P(A_k|D_i) = \frac{N_{ik}}{N_i}$$

Estimation of PASS prediction accuracy and dependency required for calculation probabilities Pa and Pi on the basis of B statistics, are the end result of the ***training procedure***, which consists in the following. According to the SAR Base, formed on the basis of the training set, for each kind of activity A_k, for each N_k active, and for each $N - N_k$ inactive compound, B statistics values are calculated. Calculations are carried out in the Leave-One-Out Cross-Validation (LOO CV), i.e., after the "exclusion" of the compound from SAR Base, for what is enough to not include it in the sum. Smooth estimations of the distribution functions $Pa(B)$ and $Pi(B)$ are based on the obtained sets of B statistics.

The probabilities Pa and Pi are both the measures of belonging to subsets of "active" and "inactive" compounds, and the probabilities of the 1st and 2nd kinds of prediction error, respectively. These two interpretations of the probabilities Pa and Pi are equivalent and can be used for understanding the results of prediction. It allows constructing different criteria for analyzing the results of prediction corresponding to the solution of specific practical problems.

An important feature of the PASS prediction algorithm is its robustness to the imperfection of information on the structure and biological activity spectrum of organic compounds in the training set. In a special study [3] we showed that halving the known information about the structure or activity of organic compounds in the training set only slightly reduces the accuracy of prediction in cross-validation. This demonstrated that the accuracy based on a LOO CV is even more rigid than for cross-validation.

Interpretation of Prediction Results

It is necessary to remember that probability *Pa* first of all reflects the similarity of molecule under prediction with the structures of molecules, which are the most typical in a sub-set of "actives" in the training set. Therefore, usually there is no direct correlation between the *Pa* values and quantitative characteristics of activities. Even active and potent compound, whose structure is not typical to the structures of "actives" from the training set, may obtain a low *Pa* value and even *Pa* < *Pi* during the prediction. This is clear from the way how the functions *Pa*(*B*) and *Pi*(*B*) are constructed: the values *Pa* for "actives" and *Pi* for "inactives" are distributed fully uniformly. Taking this into account, the following interpretation of prediction results is possible.

If, for instance, *Pa* value equals to 0.9, then for 90% of "actives" from the training set the values are less than for this compound, and only for 10% of "actives", this value is higher. If we decline the suggestion that this compound is active, we will make a wrong decision with probability 0.9.

In case if *Pa* value is less than 0.5, but *Pa* > *Pi*, for then more than half of "actives" from the training set the values are higher than for this compound. If we decline the suggestion that this compound is active, we will make

a wrong decision with probability less than 0.5. In such case, the probability to confirm this kind of activity in the experiment is small, but it will be confirmed more than 50% chances that this structure has a high novelty.

If the predicted biological activity spectrum is wide, the structure of the compound is quite simple, and does not contain peculiarities, which are responsible for the selectivity of its biological action.

If it appears that, the structure under prediction contains a few new MNA descriptors (in comparison with the descriptors from the compounds of the training set), then the structure has low similarity with any structure from the training set, and the results of prediction should be considered as very rough estimates.

The principal event used in the analysis the predicted PASS biological activity spectra is consideration of the real possibility of experimental testing. In this general recommendation is consistent study of the various predicted biological activities, from most probable to least probable.

In Figure 1 an example of the prediction of biological activity spectrum of the Topiramate is shown.

Topiramate is indicated [4] for: Treatment of Bipolar Disorder, Psychiatric Disorders (Not Specified), Treatment of Alcohol Dependency, Prophylactic Treatment of Migraine, Agents for Inflammatory Bowel Disease, Antiobesity Drugs, Antimigraine Drugs, Treatment of Cocaine Dependency, Treatment of Neuropathic Pain, Antiepileptic Drugs, Treatment of Substance Dependency, Treatment of Eating Disorders, Aid to Smoking Cessation, Treatment of Nutritional Disorders, Treatment of Cerebrovascular Diseases, Treatment of Obsessive-Compulsive Disorder (OCD). As the picture 1 indicates, in the majority of cases predicted effects by means of PASS Online correspond to the known data.

The relationship between Topiramate and drugs metabolism enzymes CYP3A4 and CYP2C19 is known, which corresponds to the prediction.

The known Topiramate’s molecular mechanisms of action include: Sodium Channel Blockers, Carbonic Anhydrase Type II Inhibitors, AMPA Antagonists, Kainate Antagonists. Topiramate as sodium channel blocker is predicted with probability Pa=0.710. Prediction points to interoperability of Topiramate and Carbonic Anhydrase Type II (Carbonic anhydrase II stimulant, Pa=0.988; Carbonic anhydrase II inhibitor, Pa=0.563), however this result does not allow to arrive at a conclusion about direction of effect (stimulation or inhibition). In such case, on which agonistic and antagonistic actions on the receptors, stimulation or inhibition of enzymes. In such cases, when predicted simultaneously agonistic or antagonistic effect on receptor stimulation or inhibition of enzymes, opening and blocking or blockade the channel, etc., a detailed study of the nature of the effect depending on the dose is required (known situations where the drug exhibits opposing effects at various doses). Perhaps this fact explains the presence of glaucoma in the prediction of activities, one of which is known to be inhibition of carbonicanhydrase II. It has been found, that the use of Topiramate induces glaucoma in some patients [6].

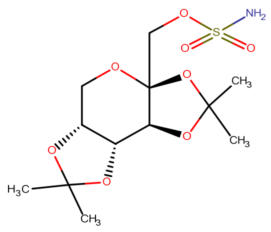

Pa	Pi	Activity
0,995	0.002	**Anticonvulsant**
0.988	0.000	**Carbonic anhydrase II stimulant**
0.954	0.001	**Antialcoholic**
0.925	0.001	**Bipolar disorder treatment**
0.923	0.002	**CYP2C19 inhibitor**
0.920	0.002	**CYP3A4 inducer**
0.914	0.002	**CYP3A inducer**
0.907	0.001	Growth stimulant
0.897	0.003	Ophthalmic drug
0.893	0.006	**CYP3A4 substrate**
0.882	0.003	**Antineurogenic pain**
0.874	0.007	**CYP3A substrate**
0.862	0.003	Antiglaucomic
0.855	0.002	**Antismoking**
0.826	0.001	Carbonic anhydrase V inhibitor
0.810	0.001	**Obsessive-compulsive disorder treatment**
0.812	0.003	**GABA receptor agonist**
0.802	0.004	**Antiepileptic**
0.731	0.001	Carbonic anhydrase IX inhibitor
0.710	0.004	**Sodium channel blocker**
0.660	0.004	Imidazoline I1 receptor agonist
0.597	0.004	**Antimigraine**
0.589	0.001	Carbonic anhydrase inhibitor
0.563	0.001	**Carbonic anhydrase II inhibitor**
0.564	0.003	**Gastric antisecretory**
0.546	0.016	**Antiobesity**
0.489	0.030	Antiallergic
0.468	0.026	CYP17 inhibitor
0.445	0.009	**Dependence treatment**
0.425	0.001	Carbonic anhydrase I inhibitor
0.463	0.048	**Analgesic**
0.434	0.042	Glucan 1,4-alpha-maltotriohydrolase inhibitor

PASS does not predict molecular mechanisms of action of Topiramate such as AMPA and Kainate Antagonists, which indicates low structural similarity of the drug with the most typical molecules that have these kinds of activity in the PASS training set.

Topiramate is also predicted to interact with GABA receptors and there are some relevant publications (e.g., [7]).

For the prediction effect of Topiramate on imidazoline receptors (Imidazoline I1 receptor agonist), and anti-allergy effects (Antiallergic) we could not find experimental evidence in the literature. It is reasonable to test in subsequent experiments these kinds of biological activities together with other forms of action of carbonic anhydrase (Carbonic anhydrase V inhibitor, Carbonic anhydrase IX inhibitor, Carbonic anhydrase I inhibitor).

PASS software provides the estimates which parts of a molecule produce positive or negative impact onto the particular activity.

Example of evaluation the contribution of each of the atoms of the Topiramate's structure for two different kinds of activity (Antiobesity и Anticonvulsant) given below:

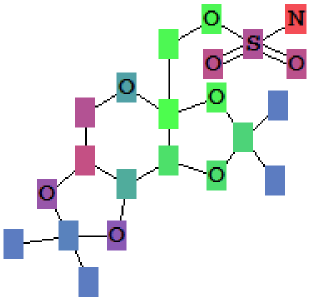

Antiobesity

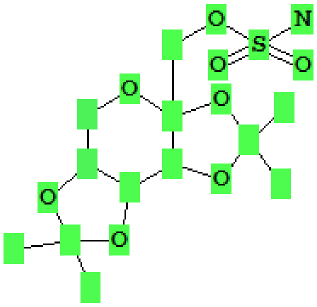

Anticonvulsant

Each of the atoms of the current structure will be colored based on the type of its contribution to the selected activity according to the following scheme:

green atom promotes activity

red atom promotes inactivity

blue atom does not generate any signal

This information can be used for directed modification of the molecular structure to enhance the desired effect and undesired attenuation mechanisms [8].

References

1 Filimonov, D.; Poroikov, V.; Borodina, Y.; Gloriozova, T. Chemical similarity assessment through multilevel neighborhoods of atoms: Definition and comparison with the other descriptors. *J. Chem. Inf. Comput. Sci.* **1999**, *39*, 666–670. https://doi.org/10.1021/ci980335o

2 Filimonov, D.A.; Poroikov, V.V. Prediction of biological activity spectra for organic compounds. *Russ. Chem. J.* **2006**, *50*, 66–75.

3 Poroikov, V.V.; Filimonov, D.A.; Borodina, Y.V.; Lagunin, A.A.; Kos, A. Robustness of biological activity spectra predicting by computer program PASS for noncongeneric sets of chemical compounds. *J. Chem. Inf. Comput. Sci.* **2000**, *40*, 1349–1355. https://doi.org/10.1021/ci000383k

4 Cortellis Drug Discovery Intelligence. Available online: https://clarivate.com/life-sciences-healthcare/cortellis/ (accessed on 4 June 2026).

5 Levy, J.; Yagev, R.; Petrova, A.; Lifshitz, T. Topiramate-induced bilateral angle-closure glaucoma. *Can. J. Ophthalmol.* **2006**, *41*, 221–225. https://doi.org/10.1139/I06-012

6 Janssen Pharmaceuticals. TOPAMAX® (topiramate) Prescribing Information. Available online: https://www.jnjlabels.com/package-insert/product-monograph/prescribing-information/TOPAMAX-pi.pdf (accessed on 4 June 2026).

7 Bandini, F.; Arena, E.; Mauro, G. Pre-orgasmic sexual headache responsive to topiramate: A case report. *Cephalalgia* **2012**, *32*, 797–798. https://doi.org/10.1177/0333102412452046

8. Wermuth, C.G. Selective optimization of side activities: The SOSA approach. Drug Discov. *Today* **2006**, *11*, 160–164. https://doi.org/10.1016/S1359-6446(05)03686-X
